# Protocol for efficient regulation of *in vitro* morphogenesis in einkorn (*Triticum monococcum* L.), a recalcitrant diploid wheat species

**DOI:** 10.1371/journal.pone.0173533

**Published:** 2017-03-08

**Authors:** Dmitry Miroshnichenko, Inna Chaban, Mariya Chernobrovkina, Sergey Dolgov

**Affiliations:** 1 Institute of Basic Biological Problems RAS, Pushchino, Moscow Region, Russian Federation; 2 Branch of Shemyakin and Ovchinnikov Institute of Bioorganic Chemistry RAS, Pushchino, Moscow Region, Russian Federation; 3 All-Russia Research Institute of Agricultural Biotechnology, Moscow, Russian Federation; United States Department of Agriculture, UNITED STATES

## Abstract

Einkorn (*Triticum monococcum* L.) is A-genome diploid wheat that has a potential to become a useful model for understanding the biology and genomics in *Triticeae*. Unfortunately, the application of modern technologies such as genetic engineering, RNAi-based gene silencing and genome editing is not available for einkorn as there is no efficient *in vitro* tissue culture and plant regeneration system. In the present study an efficient and simple protocol for plant regeneration via direct or indirect somatic embryogenesis and organogenesis has been developed. Various auxins used as sole inductors in einkorn displayed low effect for morphogenesis (0–8%) and plant regeneration (1–2 shoots per explant). The addition of Daminozide, the inhibitor of biosynthesis of gibberellins, together with auxin significantly improved the formation of morphogenic structures, especially when Dicamba (51.4%) and Picloram (56.6%) were used for combination; furthermore, the simultaneous addition of cytokinin into induction medium significantly promoted *in vitro* performance. Among the tested cytokinins, the urea-type substances, such as TDZ and CPPU were more effective than the adenine type ones, BA and Zeatin, for the regulation of morphogenesis; especially, TDZ was more effective than CPPU for shoot formation (11.73 vs. 7.04 per regenerating callus). The highest morphogenic response of 90.2% with the production of more than 10 shoots per initial explant was observed when 3.0 mg/L Dicamba, 50.0 mg/L Daminozide and 0.25 mg/L TDZ were combined together. Along with the identification of appropriate induction medium, the optimal developmental stage for einkorn was found as partially transparent immature embryo in size of around 1.0 mm. Although in the present study the critical balance between plant growth regulators was established for einkorn only, we assume that further the proposed strategy could be successfully applied to other recalcitrant cereal species and genotypes.

## Introduction

Einkorn (*Triticum monococcum* L.) is an ancient A-genome diploid wheat (2n = 2x = 14, A^m^A^m^), that was domesticated approximately 7500 BC [1]. Today it is a relic crop; however it is still growing in isolated lands of South Europe, Minor Asia, Caucasus and North Africa to provide acceptable yields on poor soils [2]. Now einkorn is increasingly used in modern wheat breeding programs as a source of novel traits such as resistance to pests and diseases, tolerance to abiotic stresses and also gains a new interest as functional foods [3]. Because of relatively small genome size, higher level of polymorphism and easy cultivation procedure einkorn also has a potential to become a useful model for understanding the biology, genomics and proteomics in *Triticeae* species and for discovery of novel genes [4]. Various transgenic technologies including RNAi-based gene silencing, tissue and time specific gene expression and chloroplast transformation may be used in diploid model for understanding the wheat genomics [5]. Unfortunately to date einkorn is regarded as recalcitrant monocotyledonous species for genetic transformation [6] and there is a need to optimize the protocol for plant regeneration in einkorn prior to attempting gene transfer.

Despite the advancements in tissue culture practices which have occurred over the past decades in tetraploid durum wheat and hexaploid bread wheat, a limited number of studies have been devoted to diploid wheats. In recent years, however, there is an increasing interest in such studies [7–9]. To date several researches demonstrated the possibility to induce *in vitro* plant regeneration from different tissues of diploid wheats including einkorn, but the achieved levels of efficiency remain too low for use in transformation experiments [10–11]. The relatively successful result for einkorn was achieved by Eudes et al. [12], who reported that a moderate number of plants (1–4 shoots per explant) could be induced without an intervening callus phase by using specific method of direct and secondary somatic embryogenesis. However, such plant regeneration efficiency is usually regarded as nonsufficient to be used for producing transgenic polyploid wheats [13–14]. Besides the method proposed by Eudes et al. [12] requires the change of as much as five culture media consisting of a high number of components (higher than the majority of currently available systems) to control adequate somatic embryo/organ development. Two recent studies reported the possibility to induce embryogenic callus from immature and mature zygotic embryos of einkorn at satisfactory level, but still the number of regenerated shoots was less than 0.5 shoots per explant [7] or there was no information concerning the number of regenerated shoots [8]. Our preliminary experiments also showed very low efficiency of embryogenesis and plant regeneration in einkorn under common protocol for cultured immature tissues [9]. In this context the development of high-performance regeneration systems for einkorn is rather critical for establishment of reliable genetic transformation procedures.

The essential prerequisite for production of transgenic plants is the availability of a target tissue including cells competent for plant regeneration. In polyploid wheats immature zygotic embryos are the most frequently and successfully used explants for plant regeneration and genetic transformation compared to other tissues [8–9, 12, 14–23]. Generally two developmentally different morphogenic pathways lead to plant regeneration from cultures of zygotic embryos: somatic embryogenesis and de novo organogenesis [24]. It is well known that somatic embryogenesis/organogenesis essentially can be subdivided into induction, maintenance, and development. In cereal tissue culture all these steps must be well executed and rely on different levels of exogenous plant growth regulators to achieve highly efficient plant regeneration. As a rule, the induction phase, that is the formation of organogenic tissue, is achieved through the treatment of explants by endogenous auxin. For the majority of bread and durum wheat genotypes it’s usually sufficient the supplement of 2–3 mg/L of 2,4-D to induce somatic embryogenesis from zygotic immature embryos [13–14, 16, 18–19, 21–22, 25–27]. Alternatively, the other synthetic auxins, such as Dicamba (DIC), Picloram (PIC) and 4-CPA can replace 2,4-D to provide the significant rising of tissue culture efficiency for specific wheat germplasms [17, 20, 23, 28–31].

Several reports however indicate that the combination of auxin with other plant growth regulators, such as cytokinins [32], abscisic acid [33], gibberellins-inhibitor [34] or ethylene-inhibitor [35] is more effective in regulation of morphogenesis. In cereals tissue culture cytokinins are not essential for callusogenesis and embryogenesis, but they can be very helpful in raising the number of regenerated plants and in reducing the time of shoot emergence when the embryogenic calli is transferred to regeneration medium [32]. For example, BA was found to promote regeneration from scutellum embryogenic structures of European bread and durum wheat cultivars [18; 28]. Improved regeneration protocols were also developed for a number of polyploid wheat varieties using different concentrations of Thidiazuron (TDZ), applied along or in combinations with other plant growth regulators [27, 31, 36–37]. Barro et al. [19] increased the regeneration capacity of callus induced from immature embryos of wheat, barley and tritordeum by addition of Zeatin (ZEA) in high concentration combined with low level of 2,4-D.

Nevertheless a number of other tools can improve morphogenic callus initiation and plant regeneration from wheat zygotic embryo tissue. Most improvements occur through stage-specific medium optimization that involves, but is not limited to, manipulation of basal salts [14], choice of sugar types or/and their combination (19; 30), addition of water potential mediators [33], organic acids [17], and some others substances. It has also been reported that the donor plant quality and proper developmental stage of explants also can promote the increasing of wheat plant regeneration [16, 22, 29].

The aim of the present study was to develop rapid and efficient regeneration protocol enabling the production of high number of plants from immature embryo cultures of einkorn. The various types of plant growth regulators (auxin, cytokinin, gibberellins inhibitor), their concentrations and combinations, as well as the developmental stage of explants were studied to achieve the efficient induction of morphogenic response. Furthermore, a histological observation was performed in order to clarify the pathways of origin and development of the resultant shoots.

## Materials and methods

### Plant material

Plants of einkorn *(T*.*monococcum* L.*)* were grown in potted soil under a 16-h photoperiod with additional lighting during the winter period for providing light intensity up to 150 μmol/m^-2^.s^-1^. Day and night temperatures in the greenhouse ranged between 18 and 25°C. Caryopses were collected 12–14 days after pollination, and subsequently they were surface-sterilized in a solution of 16.5% (v/v) commercial bleach (ACE laundry bleach) containing few drops of Tween 20 for 18 min under continuous agitation with the subsequent five-fold washing with sterile water. Immature embryos were dissected from caryopses under a stereomicroscope using sterile forceps and a scalpel and then placed scutellum-side up onto the callus induction media. Generally slightly translucent embryos of 0.75–1.5 mm in size were used, with an exception of the last experiment (the effect of developmental stage of explants) when the size of immature embryos varied from 0.5 to 2.0 mm.

### General culture media and culture conditions

Callus induction media used in this study consisted of Murashige and Skoog (MS) macro- and micro-elements [38], 0.1 mg/L thiamine HCl, 0.5 mg/L nicotinic acid, 0.5 mg/L pyridoxine HCl, 2.0 mg/L glycine, 150 mg/L L-asparagine, 3% (w/v) sucrose, and was solidified with 7 g/L agar (European type technical grade, Panreac, Spain). pH was adjusted to 5.8 before autoclaving at 121°C for 20 min. Plant-growth regulator solutions were filter sterilized with the use of Millipore filters with the diameter of 0.22 μm and afterwards were added to the autoclaved medium. In all experiments isolated immature embryos were cultured in Petri dishes for 30 days at 25°C in the dark. By the end of culture the callus induction rate was calculated.

Two subcultures, regeneration (15 days of culture) and plant development/rooting (30 days of culture), were performed to regenerate well developed green plants from induced calli. In all experiments medium consisted of MS macro- and micro-elements, 0.1 mg/L thiamine HCl, 0.5 mg/L nicotinic acid, 0.5 mg/L pyridoxine HCl, 2.0 mg/L glycine, 2% (w/v) sucrose and 7 g/L agar was used. Plant growth regulators were not added to the media, unless mentioned otherwise. To stimulate plant regeneration induced calli were first exposed for 15 days to a high light intensity (100 μmol m^–2^ s^–1^) and 16-h photoperiod at 24±2°C. Following a regeneration subculture the number of calli producing morphogenic structures was calculated. After that morphogenic calli were transferred into culture flasks under 16 hour illumination of 40 μmol m^-2^s^1^ to produce well developed plants and induce root system within 30 days. By the end of plant development/rooting subculture the percentage of regenerating calli and the number of plantlets from the regenerating calli were calculated. Rooted plants with the height of at least 5 cm were transplanted to soil as described [31] and grown under greenhouse conditions to examine the growth and the fertility.

### Effect of auxins on callus induction

In the first experiment three auxins, 3,6-dichloro-o-anisic acid (Dicamba: DIC), 4-amino-3,5,6-trichloropicolinic acid (Picloram: PIC) and 2,4-dichlorophenoxyacetic acid (2,4-D), were evaluated as the inductors of morphogenic callus. Tested concentrations of each auxin were 1, 2, 3, 4, 5 and 6 mg/L. Two replicate Petri dishes with 18–22 immature embryos each were used for one experiment per auxin treatment. That experiment was repeated at least three times.

### Effect of Daminozide on callus induction

In the second experiment the effect of succinic mono-N,N-dimethylhydrazide (Daminozide: DAM) in combination with 3 mg/L DIC was studied. Five concentrations, 0, 25, 50, 100 and 150 mg/L, of DAM were analyzed. For one experiment, two replicate Petri dishes, each with 25–30 immature embryos, were used per DAM treatment. The experiment was repeated four times.

### Effect of cytokinins on plant regeneration from induced callus

The experiment was designed to promote plant regeneration from calli that were induced on callus induction medium supplemented with 3 mg/L DIC and 50 mg/L DAM. In that experiment 276 induced morphogenic calli were incubated for 15 days in Perti dishes contained the regeneration media supplemented with 0.5 or 1.0 mg/L 6-Benzylaminopurine (BA), thidiazuron (TDZ), or zeatin (ZEA). After that the morphogenic calli were transferred into culture flasks containing the medium lack of phytohormones, for 30 days. For single experiment, two culture jars, with 9–10 morphogenic calli each, were used within cytokinin concentration. The experiment was double repeated.

### The combined effect of PGRs combination (auxin + cytokinin + Daminozide)

Two independent experiments were carried out. Initially the callus induction medium containing 3 mg/L DIC and 50 mg/L DAM was supplemented either with BA, TDZ, ZEA or N-(2-Chloro-4-pyridyl)-N-phenylurea (CPPU). Tested concentrations of each cytokinin were 0.125, 0.25, 0.5, 1.0 and 1.5 mg/L. For the first experiment, two or three replicate Petri dishes, with 20–25 immature embryos each, were used per cytokinin treatment. The experiment was repeated at least twice. In another experiment the auxins 2,4-D, DIC and PIC at concentration of 3 mg/L were combined either with 50 mg/L DAM alone or both with 50 mg/L DAM and 0.25 mg/L TDZ in callus induction medium. For one experiment, two replicate Petri dishes, each with 25–26 immature embryos, were used per cytokinin treatment. The experiment was repeated three times.

### Effect of developmental stage of explants

In order to estimate the effect of the developmental stage of immature embryos, small-sized (0.5–1.0mm, completely transparent), intermediate-sized (1.1–1.5mm, partially transparent), and large-sized (1.6–2.0 mm, non transparent) embryos were isolated from immature caryopses. Isolated embryos of different sizes were placed onto three callus induction media with different plant growth regulators composition: 3 mg/L DIC; 3 mg/L DIC and 50 mg/L DAM; 3 mg/L DIC and 50 mg/L DAM and 0.25 mg/L TDZ. For one experiment, two or three replicate Petri dishes, each with 20–22 immature embryos, were used per explant size. The experiment was repeated at least twice.

### Histological analysis

The whole calli or their fragments were fixed in 2.5% glutaraldehyde (Merck, Germany) dissolved in 0.1 M phosphate buffer (pH 7.2) supplemented with 1.5% sucrose. Plant material was washed and postfixed in 1% OsO4, dehydrated in ethanol at increasing concentrations (30, 50, 70, 96, 100%) and, subsequently, in propylene oxide (Fluca, Germany), and then embedded in a mix of the epoxide resin Epon-812 and Araldite (Merck, Germany). For light microscopy, semifine (1–2 um) sections were prepared using glass knifes and ultramicrotome LKB-V (LKB, Sweden), placed on sample slides, stained with 0.1% methylene blue water solution (Merck, Germany), and embedded in epoxide resin. Samples were studied and photographed with the use of Olympus BX51 microscope (Olympus, Japan) equipped with Color Viev II camera (Soft Imaging System, Germany).

### Statistical analysis

The identical and independent experiments were performed for every treatments. Data were collected on a per plate/jar basis. Сallus induction frequency (%) was calculated as a number of callus producing explants to a number of initial explants × (multiplied by) 100. The rate of precocious germination (%) was calculated as a number of germinating embryos to a number of cultured embryos × 100. The frequency of morphogenic callus formation (%) was calculated as a number of calli producing at least one plain or nodular structure to a number of callusing embryos × 100. A clearly differentiated structure with at least one emerged plantlet longer than 1 cm was scored as a regenerated plant. Callus with at least one regenerated plant was counted as a regenerating calli. The regeneration capacity (%) was calculated as a number of regenerable calli to a number of callusing embryos × 100. The statistical significance of differences (*p*<0.05) between groups was tested using one or two way analysis of variance (ANOVA). Mean separation and LSD grouping was achieved by Duncan’s multiple range test at *p* < 0.05 using the same software. The statistical analysis was conducted by means of Statistica10 software.

## Results

### Effect of auxin

Regardless the efficient callus induction (90–99%) tested levels of DIC, PIC and 2,4 D displayed low effect for improving somatic embryogenesis and plant regeneration (S1 Table). Analysis of variance did not reveal significant effect of auxin type on the frequency of morphogenic callus formation. The average number of shoots was near the equal for the most of auxin treatments (1–2 shoots per 10 callusing embryo). Exception was noticed for high 2,4-D and low PIC concentrations, when cultures were not able to regenerate plant at all. The adding of DIC stimulated slightly better formation of morphogenic structures (2.15–7.76%) than PIC (1.59–4.90%) and 2,4-D (0.0–4.14%). For this reason DIC with a dosage of 3 mg/L was chosen for the subsequent experiments aimed to increase the morphogenic callus production by supplementation of DAM.

### Effect of Daminozide

**Fig 1:**
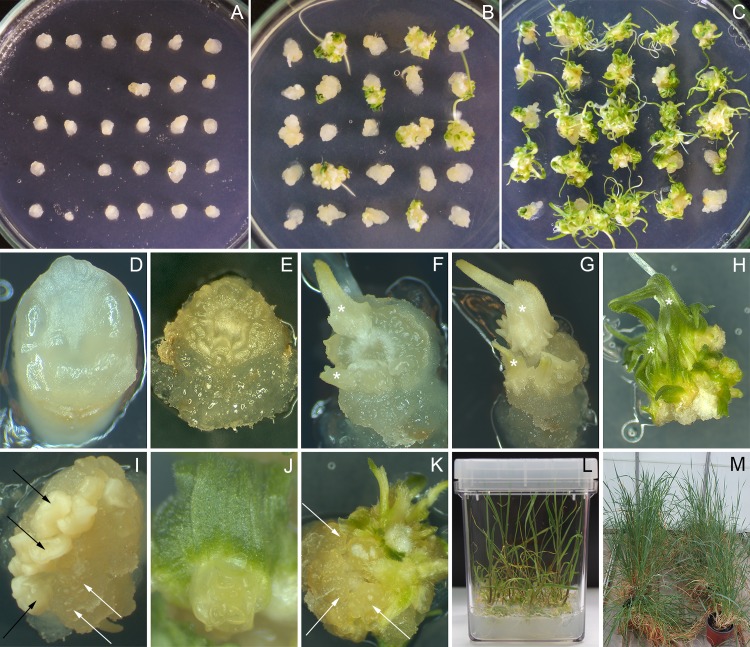
Morphological aspects of morphogenesis and plant regeneration in *Triticum monococcum* L. **(A-C).** Effect of PGRs on the regenerable callus induction from immature embryos on the medium supplemented with 3 mg/L DIC (A), 3 mg/L DIC and 25 mg/L DAM (B), 3 mg/L DIC, 50 mg/L DAM and 0.25 mg/L TDZ (C), 6 weeks of culture. (D-H, J-K) Details of morphogenesis in immature embryo cultures induced on the medium supplemented with 3 mg/L DIC, 50 mg/L DAM and 0.25 mg/L TDZ; (D) swelling at the inner part of cultured immature scutellum, 7 days of culture; (E) appearance of nodular structures on the scutellum, 14 days of culture; (F) formation of leaf-like structures (asterisks), 20 days of culture, and (G) their development by the end of the callus induction phase; (H) differentiation of multiple plantlets on regeneration medium under 16 h of photoperiod, 40 days of culture; (J) shoot apices formation at the base of leaf-like structure on regeneration medium, 50 days of culture; (K) pro-morphogenic granular masses (black arrows) appeared on the same explant concomitantly with the development of plantlets and leaf-like structures (asterisks), 35 days of culture. (I) Callus induced on the medium supplemented with 3 mg/L DIC and 50 mg/L DAM displaying the formation of non translucent scutellum-like (white arrows) and translucent off-white nodular (black arrows) morphotypes, 30 days of culture. (L) In vitro plant regeneration from morphogenic callus, 25 days of culture on medium lack of growth regulators under 16-hour photoperiod. (M) Mature regenerated plants growing under greenhouse conditions.

Table 1 summarizes the effects of DAM on the efficiency of *in vitro* tissue culture of einkorn. Analysis of variance revealed that the presence of DAM in induction medium had significant positive effect on the efficiency of morphogenic callus induction, as well as on the frequency of regeneration from callus even at the lowest tested concentration. The most of einkorn explants formed small soft non-regenerating amorphous callus without DAM (Fig 1A); wherein the rate of morphogenesis was only 7.5%. The strong stimulatory effect of DAM was found at a level of 50–100 mg/L when more than a half of cultivated explants produced morphogenic structures (Fig 1B). Two types of morphogenesis were found discovered (Fig 1I). Within 2–3 weeks the formation of bright yellow-white, non translucent nodular-organized structures were observed. The higher concentration of DAM was applied the greater portion of white non translucent structures emerged. By the end of cultivation, the formation of another morphotype consisting of translucent off-white nodular callus was also revealed (Fig 1I).

**Table 1:** The influence of DAM concentrations on morphogenesis and plant regeneration from immature embryos of einkorn (*Tritcum monococcum* L.)

DAM concentration (mg/L)	Callus induction (%)	Morphogenic callus formation (%)	Percentage of regenerating calli (%)	No. of plantlets per regenerating calli	No. of plantlets per callus
0	94.15 a	7.45 c	4.35 c	4.71 a	0.21 c
25	98.33 a	27.12 b	20.90 b	5.73 a	1.20 b
50	96.02 a	52.66 a	34.91 a	5.61 a	1.96 a
100	94.25 a	52.44 a	33.54 a	5.13 a	1.72 ab
150	99.42 a	48.54 a	29.24 ab	5.26 a	1.54 ab

All cultures were initiated within 30 days on callus induction medium supplemented with 3 mg/L DIC in combination with different concentrations of DAM with the subsequent two subcultivations for 15 and 30 days on regeneration medium lack of PGRs. Means having the same letter in the column has no significant differences according to Duncan’s multiple range test (*P* < 0.05).

A few days after transferring to regeneration medium, and accommodation to light, morphogenic calli formed green sectors started to convert into visible plantlets within 10–15 days. The percentage of regenerating scutellar calli varied depending on DAM treatment; the maximum rate of 34–35% was scored at 50–100 mg/L. In average 5–6 green plantlets developed per one regenerating callus of einkorn (Table 1). Analysis of variance revealed that DAM had no significant effect on this parameter and near the same number of plants was scored at the control medium lack of DAM (Table 1). Despite the fact that the third of morphogenic structures induced on the media supplemented with 50 mg/L of DAM was unable to develop plantlets, given concentration was the most effective in promoting plant regeneration in cultivated zygotic einkorn embryos. It resulted in 10 fold average number increasing of regenerated plants per callusing zygotic embryo (1.96 plantlets) as compared with the control medium (0.21 plantlets).

### Effect of cytokinins on plant regeneration from morphogenic calli

Results obtained from the previous experiment indicated that a significant portion of non translucent morphogenic structures induced in the presence of DAM were not able to form plantlets after transferring to hormone-free regeneration medium. In the present experiment calli with morphogenic structures induced in the presence of 50 m/L DAM and 3 mg/L DIC were transferred onto regeneration medium supplemented with various cytokinins, in order to stimulate plant regeneration. The results of the experiment are shown in Table 2. The statistical analysis (ANOVA) revealed that the presence of BA or ZEA in regeneration medium had no stimulatory effect on plantlet formation. In contrast, the addition of TDZ had a significant influence on the development of plantlets, especially at the higher concentration. In average 85.95% of morphogenic calli subcultured on the regeneration media containing TDZ successfully regenerated plants. Herewith the percentage of regenerating callus was near 70% on the media supplemented with the same concentrations of BA or ZEA, as well as on the media lack of growth regulators.

**Table 2:** The influence of cytokinin type on plant regeneration from morphogenic calluses of einkorn (*Tritcum monococcum* L.).

Cytokinin	Concentration (mg/L)	Regeneration capacity (%)	No. of plantlets per regenerating calli	No. of plantlets per callus
Hormone-free	-	70.0 bc	5.46 b	1.95 b
BA	0.5	72.5 bc	5.07 b	1.88 b
	1.0	67.5 c	5.78 b	1.99 b
	mean	70.0 ns	5.42 ns	1.94 ns
TDZ	0.5	82.1 ab	6.88 b	2.81 b
	1.0	89.7 a	11.37 a	5.08 a
	mean	85.9*	9.12*	3.94*
ZEA	0.5	64.1 c	5.60 b	1.79 b
	1.0	76.9 abc	6.03 b	2.31 b
	mean	70.5 ns	5.82 ns	2.05 ns

276 morphogenic calli were initiated on callus induction medium supplemented with 3 mg/L DIC and 50 mg/L DAM and then cultivated for 15 days on regeneration medium supplemented with cytokinin in different concentrations with the subsequent subcultivation for 30 days on regeneration medium lack of PGRs. Means having the same letter in the column has no significant differences according to Duncan’s multiple range test (*P* < 0.05)

* indicates significance level at *P* < 0.05 based on LSD test between the control hormone-free medium and the medium containing specific cytokinin, **ns** non significant at *P* < 0.05.

TDZ treatment stimulated the multiple formations of regenerated plants in einkorn; the number of plants reached 11.37 plantlets per regenerating calli at TDZ concentration of 1 mg/L. At the same time the tendency to increase the number of shoots in regeneration media supplemented with the lower TDZ concentration was not proved statistically. When morphogenic calli were cultivated on the medium containing BA or ZEA no stimulating effect was found due to the fact that the same number of shoots (5–6 plantlets per regenerating calli in average) were scored on the medium lack of growth regulators. Regarding the number of shoots per initiated callus, TDZ demonstrated a tendency to improve this parameter at both tested concentrations (in average 3.94 shoots vs. 1.95 shoots in hormone-free medium). At the same time, according to statistical analysis, only the incorporation of 1.0 mg/L TDZ into regeneration media has demonstrated a strong promoting effect, resulted in production of about 5 green plants per initial explant, that was two and a half times higher when using the hormone-free medium (Table 2).

### The combined effect of PGRs combination (auxin + cytokinin + Daminozide)

#### The effect of cytokinin

Despite the improvement of the regeneration ability during the previous experiment by incorporation of cytokinin at the regeneration stage, the main challenging problem, namely poor rate of morphogenic structures induction, still remained for einkorn tissue culture. In this study the stimulatory effect of various cytokines, including BA, ZEA, TDZ and CPPU, used in combination with DIC and DAM, was assessed to stimulate morphogenesis. Both the cytokinin type and its concentration significantly influenced the parameters of einkorn tissue culture of (Table 3). A clear positive effect on the rate of morphogenic callus formation has been found when diphenylurea derivatives were added into callus induction media. Significant increase in morphogenic and regenerable callus induction was observed on the medium supplemented with TDZ (81.08% and 79.65%) and CPPU (72.98% and 68.65%). Simultaneously, purine derivative structures, such as BA and ZEA, generally had no statistically significant advances in stimulation of morphogenic/regenerating callus production as well as in improvement of an average number of regenerated plants in comparison with the control medium. The only exception was found for the highest BA concentration when the induced morphogenic calli displayed higher regeneration capacity (53.66%) than that of control (32.19%), though the overall culture efficiency in terms of shoots per callusing embryo changed insignificantly due to reduction of callus production at 1.5 mg/L BA. The similar suppression of callus formation was observed at the highest concentration of ZEA and CPPU. The variation of TDZ level had no significant influence on the callus induction rate. At the same time higher concentrations of TDZ (1.0–1.5 mg/L) accelerated the browning of calli and negatively affected the morphogenesis rate. Actually the best result for morphogenic callus production (88–91%) was obtained when the immature embryos of einkorn had been cultivated in the presence of 0.125–0.250 mg/L TDZ (Fig 1C). At these concentrations the percentage of regenerating calli reached 86–90%, that was significantly higher in comparison with the medium lack of cytokinin (51.37%).

On the other hand, the analysis of variance did not reveal the significant differences in the rate of morphogenic/regenerable calli formation between various concentrations of TDZ (0.125–0.250 mg/L) and CPPU (0.5–1.0 mg/L), since at least 80% of callusing immature embryos produced morphogenic structures and shoots (Table 3). Herewith there was significantly more number of shoots formed in the presence of TDZ, with the average of 11.73 shoots per regenerating calli, than on the medium supplemented with CPPU, where the mean number of shoots was 7.04. In any case, according to LSD test both diphenylurea derivatives were more effective in stimulation of regeneration than BA and ZEA (Table 3). It should be noted, that the media supplemented with higher TDZ concentrations (1.0–1.5 mg/L) caused abnormal development in regenerated plants, e.g. the shoot length and thickness were inferior to those on the medium with lower concentrations; moreover the symptom of vitrification was observed.

**Table 3:** The influence of various cytokinin concentrations on morphogenesis and plant regeneration from immature embryos of einkorn (*Tritcum monococcum* L.).

Cytokinin	Concentration (mg/L)	Callus induction (%)	Morphogenic callus formation (%)	Percentage of regenerating calli (%)	No. of plantlets per regenerating calli	No. of plantlets per callus
Control	0,000	98.65 a	51.37 cd	32.19 d	5.71 efg	1.82 ef
BA	0.125	99.20 a	43.55 d	31.45 d	5.04 fg	1.60 ef
	0.250	98.00 a	50.00 cd	30.61 d	4.65 fg	1.59 ef
	0.500	96.00 a	56.67 cd	44.17 bcd	5.66 efg	2.50 ef
	1.000	88.80 abc	57.66 cd	40.54 bcd	5.49 efg	2.23 ef
	1.500	78.85 bc	68.29 bc	53.66 b	5.07 fg	2.74 ef
	**mean**	**92.62 ns**	**54.85 ns**	**39.89 ns**	**5.18 ns**	**2.15 ns**
CPU	0.125	96.30 a	61.54 cd	53.85 b	6.27 ef	3.38 de
	0.250	96.32 a	80.92 ab	77.86 a	7.65 de	5.95 bc
	0.500	91.91 ab	82.40 ab	80.00 a	7.80 de	6.24 bc
	1.000	81.48 bc	81.82 ab	79.09 a	6.91 ef	5.46 bc
	1.500	76.30 c	58.25 cd	52.43 bc	6.56 ef	3.44 de
	**mean**	88.48*	72.98*	68.65*	7.04*	**4.89** *
TDZ	0.125	98.08 a	88.24 a	86.27 a	9.30 cd	8.02 ab
	0.250	96.06 a	90.98 a	90.16 a	11.17 bc	10.07 a
	0.500	89.43 abc	81.82 ab	80.91 a	12.47 ab	10.10 a
	1.000	93.08 ab	80.99 ab	80.17 a	11.45 bc	9.18 a
	1.500	93.27 ab	60.82 cd	57.73 b	14.27 a	8.24 ab
	**mean**	**93.80 ns**	81.08*	79.65**	11.73**	**9.12** **
ZEA	0.125	91.00 ab	50.55 cd	35.16 cd	4.69 fg	1.65 ef
	0.250	91.84 ab	50.00 cd	32.22 d	4.48 fg	1.44 ef
	0.500	91.00 ab	54.95 cd	35.16 cd	5.16 fg	1.81 ef
	1.000	95.92 a	61.70 cd	46.81 bcd	5.23 fg	2.45 ef
	1.500	76.15 c	24.10 e	9.64 e	3.88 g	0.37 f
	**mean**	89.20*	**48.27 ns**	**31.79 ns**	**4.69 ns**	**1.54 ns**

All cultures were initiated within 30 days on callus induction medium containing 3 mg/L DIC and 50 mg/L DAM (regarded as control) in addition supplemented with different concentrations of cytokinins as followed by two subcultivations for 15 and 30 days on regeneration medium lack of PGRs. Means having the same letter in the column has no significant differences according to Duncan’s multiple range test (*P* < 0.05)

* and ** indicate significance level at *P* < 0.05 or *P* < 0.01 based on LSD test between the control medium and the medium containing specific cytokinin, **ns** non significant at *P* < 0.05.

In general, the formation of visible morphogenic structures on einkorn explants was observed after 10–15 days of cultivation at optimal TDZ and CPPU concentrations (Fig 1E); in the absence of cytokinin the development of morphogenic structures occurred not earlier than 20 days after initiation. The first leafy-like structures were formed during the third week of culture on TDZ and CPPU containing medium (Fig 1F), then they could be easily expanded into shoot-like structures till to the end of initiation culture (Fig 1G). Moreover the presence of urea-type cytokinin caused the formation of first plantlets as early as 7–10 days after the transfer onto regeneration medium (Fig 1H); whereas the morphogenic calli induced in the presence of auxin and DAM developed plantlets not earlier than two weeks later the second transfer onto regeneration medium in culture jars.

Taking into account the rate of morphogenic callus formation, the number and the quality of regenerated plants we suggested that the range of TDZ concentration around 0.25–0.50 mg/L was appropriate for combination with DIC and DAM. It made possible to produce 10.1 shoots per callusing zygotic embryo in average, that is far exceeding 1.8 shoots resulted from the control medium supplemented with auxin and DAM only (Table 3). Regenerated shoots (Fig 1L) with the prominent roots were transferred into soil and thereafter grown in greenhouse. A great number of plantlets made impossible to transfer all of them; therefore, random samples were taken for the observation of any abnormalities. All plantlets which had been transferred to soil, survived under greenhouse conditions. Within 4–6 months they grew to morphologically normal and fertile plants (Fig 1M).

### The effect of auxin

Considering the presented above results, in the next experiment the effect of various auxins was reassessed to find out if the combined effect of DAM and urea-type cytokinin would be stable if the differing types of synthetic auxin should be used in combination. 2,4-D, DIC and PIC were combined in callus induction medium either with DAM only or with DAM and TDZ (Table 4). Both combinations showed the significant improvement of morphogenic callus formation and regeneration capacity in einkorn. According to ANOVA analysis, in that case the type of auxin-like substances had a certain influence on morphogenic response as compared to the previous experiment presented in S1 Table. When the auxins were combined with DAM, the efficiency of morphogenic callus formation from the cultures that were derived from the explants grown on the PIC-containing medium was more than twice as efficient as morphogenesis from cultures induced on 2,4-D; almost the same response was observed on the medium contained either PIC or DIC (Table 4). However PIC showed a better ability to stimulate regeneration from the induced morphogenic calli (46.05%, 6.9 plant per regenerating calli) than DIC (34.10%, 5.6 plants per regenerating calli) and, especially, 2,4-D (18.18%, 4.4 plants per regenerating calli).

**Table 4:** The effect of PGRs combinations added to induction medium on morphogenesis and plant regeneration from immature embryos of einkorn (*Tritcum monococcum* L.).

Plant growth regulators (mg/L)					Callus induction (%)	Morphogenic callus formation (%)	Percentage of regenerating calli (%)	No. of plantlets per regenerating calli	No. of plantlets per callus
2,4-D	DIC	PIC	DAM	TDZ					
3.0			50		100.0 a	23.38 e	18.18 e	4.36 d	0.79 e
3.0			50	0.25	100.0 a	67.11 bc	63.16 b	9.52 b	6.01 b
	3.0		50		96.73 b	51.36 d	33.81 d	5.59 d	1.91 d
	3.0		50	0.25	98.03 b	91.21 a	89.85 a	11.85 a	10.64 a
		3.0	50		100.0 a	56.58 cd	46.05 c	6.91 c	3.18 c
		3.0	50	0.25	100.0 a	71.05 b	68.42 b	9.63 b	6.59 b

All cultures were initiated within 30 days on callus induction medium with the subsequent two subcultivations for 15 and 30 days on regeneration medium lack of PGRs. Means having the same letter in the column has no significant differences according to Duncan’s multiple range test (P < 0.05).

At simultaneous addition of TDZ and DAM into the culture medium, the highest combined effect in einkorn was found on DIC-containing medium; wherein the frequency of the morphogenic and regenerable callus induction (91.21% and 89.85%) as well as the number of regenerated plantlets (11.85 plants per regenerable calli) was similar to the data of the previous experiment (Table 3). PIC and 2,4-D demonstrated a lower effect as compared with DIC and induced near the same morphogenic callus production (68.4% vs. 63.2%) and plant regeneration (9.63 vs. 9.52 plantlets per regeneration calli). Regardless the applied auxin-like substances the combination with TDZ and DAM was proved for significant rise of einkorn tissue culture efficiency in any event as compared with the medium lack of cytokinin (Table 4). Nevertheless, callus induction media supplemented with DIC plus DAM and TDZ promoted formation of a higher number of plants per callus (10.64 plantlets), than PIC (6.59 plantlets) and 2,4-D (6.01 plantlets).

### Effect of developmental stage of explants

In the latest experiment, we divided the isolated immature zygotic embryos in three sizes: small-sized (0.5–1.0 mm, completely transparent), intermediate-sized (1.1–1.5 mm, partially transparent), and large-sized (1.6–2.0 mm, non transparent) in order to estimate the effect of the developmental stage of explants on the efficiency of morphogenic callus induction and plant regeneration. In einkorn the response of the immature embryos with different size is largely depends on the PGRs content in callus induction media (Table 5). The small-sized explants were more likely to produce the morphogenic structures (10.00%) as compared to the intermediate-sized (6.06%) and, especially to the big-sized explants (1.23%), if the medium was supplemented with the auxin only. In contrast, on the medium supplemented with auxin and DAM, the intermediate- and big-sized explants proved to be the more efficient in terms of morphogenesis (53.78% and 50.00%, respectively), than the small ones (35.19%). All of the cultivated small-sized explants developed morphogenic structures on callus induction medium supplemented with three PGRs, giving the maximum rate of induction (100.0%). Nevertheless, small-sized embryos had a lower ability to regenerate plants (9.63 plantlets per regeneration calli) than intermediate- and big-sized ones (12.52 and 10.42 plantlets per regenerating calli, respectively).

**Table 5:** The influence of immature embryo size on morphogenesis and plant regeneration from immature embryos of einkorn (*Tritcum monococcum* L.).

Embryo size	Plant growth regulators (mg/L)			Precocious germination rate (%)	Callus induction (%)	Morphogenic callus formation (%)	Percentage of regenerating calli (%)	No. of plantlets per regenerating calli	No. of plantlets per callus
	DIC	DAM	TDZ						
Small	3.0	-	-	5.6 d	100.0	10.00 e	10.00 e	2.44 d	0.24 f
0.5–1.0 mm	3.0	50.0	-	5.1 d	91.53	35.19 d	20.37 d	3.32 cd	0.68 ef
	3.0	50.0	0.25	3.7 d	93.75	100.0 a	100.0 a	9.63 b	9.63 b
Intermediate	3.0	-	-	7.1 d	100.0	6.06 e	3.03 e	3.33 cd	0.10 f
1.1–1.5 mm	3.0	50.0	-	24.2 c	95.97	53.78 c	38.66 c	5.24 c	2.03 d
	3.0	50.0	0.25	39.0 b	100.0	91.43 ab	89.52 b	12.52 a	11.21 a
Large	3.0	-	-	25.6 c	98.78	1.23 e	1.23 e	4.00 cd	0.05 f
1.6–2.0 mm	3.0	50.0	-	66.3 a	100.0	50.00 c	32.50 c	4.38 cd	1.43 de
	3.0	50.0	0.25	69.1 a	100.0	82.72 b	81.48 b	10.42 ab	8.49 c

All cultures were initiated within 30 days on callus induction medium with the subsequent two subcultivations for 15 and 30 days on regeneration medium lack of PGRs. Means having the same letter in the column has no significant differences according to Duncan’s multiple range test (P < 0.05).

The rate of the cultured immature embryos precocious germination raised along with the increase of their size. Irrespective of the employed callus induction medium the lowest rate of precocious germination (3.7–5.6%) was observed at the small-sized embryos, while the highest germination ability was observed when the big-sized somatic embryos were involved. Moreover the presence of DAM in the callus induction medium significantly promoted the precocious germination in both intermediate- and big-sized explants, and did not affect the germination ability of the small-sized embryos. Since developing embryo axes were removed from germinating explants during the callus induction phase, high germination ability had no significant effect on callusing/morphogenic response. Nevertheless, there was significant effect of explant developmental stage on the plant yield per one callusing embryo, especially when three PGRs were used to stimulate morphogenic callus induction. In that case the intermediate-sized explants produced more shoots per callusing embryo (11.21 plantlets) than the younger or older ones (9.63 and 8.49 plantlets, respectively). The same tendency was observed on the medium supplemented both with auxin and DAM, while on the medium supplemented with auxin only the effect of developmental stage was not prominent due to extremely low number of induced morphogenic calli.

### Morphological and histological observations

**Fig 2:**
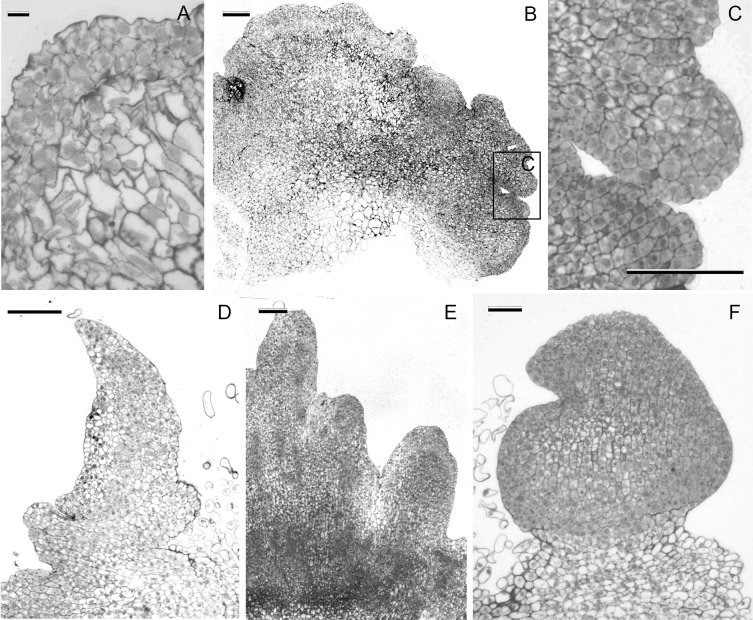
Histological study of morphogenesis in immature embryo-derived culture of *Triticum monococcum* L. on the medium supplemented with 3 mg/L DIC, 0.25 mg/L TDZ and 50 mg/L DAM. (A) Initial divisions of peripheral cells with dense cytoplasm and big nuclei in the contrast to cells deeper in the scutellar tissue that remained vacuolated, 7 days of culture, *bar* 20 μm. (B) Nodular structures progressed from active cell divisions of the inner part of cultured scutellum, 15 days of culture, *bar* 200 μm. (C) Details of nodular structure showing both pericline and anticline divisions of isodiametric cytoplasm-rich cells, *bar* 100 μm. (D) Longitudinal section of leafy-like structures presenting a connection with the original tissue of scutellum, 19 days of culture, *bar* 200 μm. (E) The axis of shoot primordia vascularly connected with the base of leaf-like structure, 40 days of culture, *bar* 200 μm. (F) Direct development of embryo-like structure without vascular connection with the original tissue, 17 days of culture, *bar* 100 μm.

The massive cell proliferation at the inner part of cultured einkorn immature scutellum was observed during the first week of cultivation on the medium contained three PGRs (Fig 1D). Those cells presented dense cytoplasm and large nuclei, in contrast to cells located deeper in the tissue that remained vacuolated (Fig 2A). By the end of the fortnight cultivation these cell divisions of the epithelial and sub-epithelial layers evolved to form nodular compact structures (Fig 1E, Fig 2B) of highly dividing isodiametric cells (Fig 2C). A part of such structures started to form leafy-like structures (Fig 1F) during the second week of culture on the medium supplemented with diphenylurea derivatives. The analysis of the anatomy of the leaf-like structures produced on the explants within 20 and 30 days cultivation confirmed the organogenic nature of the regeneration process (Fig 2D). Afterwards those structures elongated and could be observed by the naked eye since the 18–22 day from the initiation of culture (Fig 1G). By the end of the callus induction phase as well as within the first two weeks of cultivation on regeneration medium, shoot apices occurred to differentiate from extensive meristematic zones at the base of the leaf-like structures (Fig 1J and Fig 2E).

**Fig 3:**
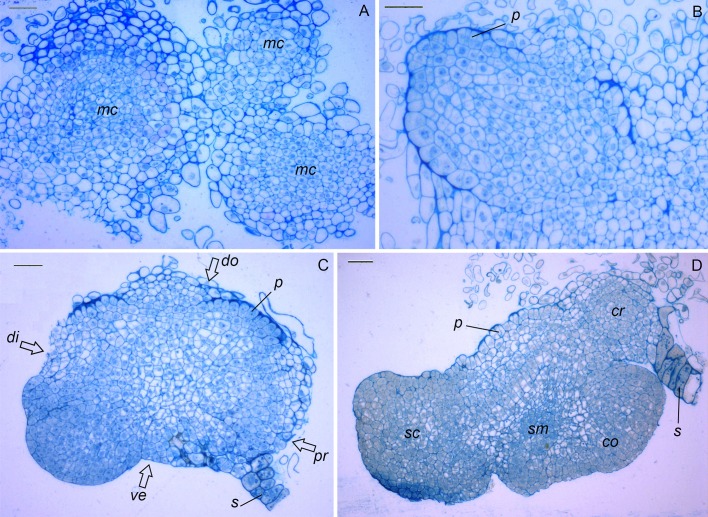
Histology of *Triticum monococuum* L. somatic embryos at different stages of development. (A) Transversal section of meristematic centers delineated from the surrounding non-organized, dispersed and vacuolated cells, *bar* 100 μm. (B) Thickened cell walls proembryo arising from friable callus tissue, *bar* 50 μm. (C) Section of globular somatic embryo with suspensor displaying transition to bilateral symmetrical orientation (open arrows), *bar* 100 μm. (D) Longitudinal section of somatic embryo at a later developmental stage showing clear bilateral symmetrical orientation, *bar* 100 μm. *p*–protoderm, *sm*–shoot meristem, *cr*–coleorhizae, *co–*coleoptile, *sc*.–scutellum, *s*–suspencer, *ve*–ventral; *do*–dorsal; *di*–distal; *pr*–proximal;

At the same time, starting from the second week of cultivation the appearance of primary embryoids-like structures in the same cultures was found. At the early stage of development the embryo was covered with protodermis and consisted of zones of dividing cells with dense cytoplasm and small vacuoles and distinguished by the absence of vascular connection with the original tissue at basal side (Fig 2F). In between 20 and 40 days of culture the callus growth progressed throughout the different parts of cultured explant, and pro-morphogenic masses could be easily distinguished both on their granular appearance, and by translucent color (Fig 1K). They had organized structures with a thickened cell wall (Fig 3A). The appearance of these early stages was soon followed by the formation of globular-shaped embryos (Fig 3B). The appearance of globular structures was coupled with the concomitant development of the protoderm, the outermost layer of a developing embryo. The protoderm was hard to distinguish at the early globular stages (Fig 3B), but it was more defined at the later stages (Fig 3C and 3D). During the transition from somatic pro-embryonic structure to globular embryo, a multicellular suspensor-like structure developed, connecting the globular embryos to the callus (Fig 3C and 3D). Those globular embryos gradually established their polarity and then converted into plantlets after transferring onto regeneration medium between the fifth and sixth week of culture (Fig 1L).

**Fig 4:**
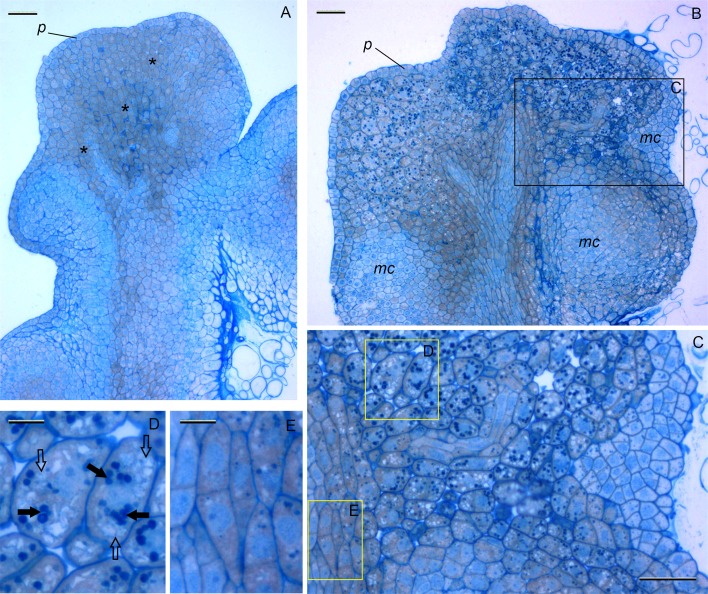
Histological analysis of hard white scutellum-like structures of *Triticum monococcum* L. induced on the medium supplemented with 3 mg/L DIC and 50 mg/L DAM. (A) Section from developing scutellum-like structures bordered by well-developed protoderm (the outer unicellular layer) at an early stage of the ‘storage-like’ differentiation (asterisks), 15 days of culture, *bar* 100 μm. (B) General view of the entire longitudinal section from the hard scutellum-like structures at the end of callus induction phase, 25 days of culture, *bar* 100 μm. (C) Detailed view from the part of longitudinal section from the scutellum-like structures, the presence of cells with various shapes and differentiation is occurred, *bar* 50 μm. (D) The presence of vacuolated big cell types with the larger intracellular spaces, starch granules (closed arrow) and stock substances on the vacuoles surfaces (open arrow), *bar* 20 μm. (E) The presence of small granules concentrated in cytoplasm along the cell walls, *bar* 20 μm. *p*–protoderm, *m*.*c*.–meristematic centers.

In contrast, the direct shoot-like structures formation from einkorn explants was never observed without cytokinin (when only auxin and DAM were added to the induction medium). The formation of non-translucent nodular structures was observed during the two to three weeks of culture predominate on this medium. Histological analysis of those hard white scutelum-like structures revealed that they were covered with a clear protoderm and had a bunch of non-organized cells proliferation with a high cytoplasm density (Fig 4A). However, further development of these structures became disturbed after second week of culture. Detailed view has found an appearance of cells with larger intracellular spaces, with numerous starch granules in cytoplasm and with distinct vacuoles (Fig 4B, 4C and 4D). In some cells the accumulation of stock substances was visible on the vacuoles surfaces (Fig 4C and 4D); the numerous small granules, presumably lipids, were concentrated in cytoplasm along the cell walls as well (Fig 4C and 4E). At the same time the areas with actively dividing cells without any ‘storage-like’ activities were observed (Fig 4B and 4C). Similarly to the cultures produced on the medium supplemented with three PGRs, new secondary proembryo units were formed more frequently on the surfaces of callus by the end of callus induction phase (Fig 1I). When the explants with non-translucent structures and translucent nodular units were removed from the induction medium supplemented with DAM with the subsequent cultivation on the regeneration medium, they were able to form numerous green sectors, and started to grow into plantlets under light in two-three weeks after their transferring (Fig 1B).

## Discussion

The somatic embryogenesis has been described as the most common pathway of plant regeneration in cultures of tetraploid and hexaploid *Triticum* sp. [39]. In cereals, however, plant regeneration can also occur through organogenesis and concomitantly through both somatic embryogenesis and organogenesis [15, 25, 40–42]. The proper identification of developmental pathways in wheat and other cereals was complicated as it was difficult to trace in detail the earliest stages of embryo and callus plant regeneration, while the induced embryos are often macroscopically similar to shoots or leaves [39–40]. In the present report, the morphological and histological evidence for the simultaneous occurrence of both somatic embryogenesis and organogenesis has been found for embryo-derived culture of diploid einkorn wheat. Herewith it was found that the combinations of PGRs used for regulation of morphogenic response, significantly influenced the type of pathway of plant regeneration and its efficiency.

PGRs are the key substances for induction of plant morphogenic response *in vitro*. Our preliminary experiments had demonstrated that diploid einkorn displayed very low efficiency of embryogenesis and plant regeneration under conventional protocol, when immature tissues were cultivated for 4 week on the MS media containing 2 mg/L 2,4-D under darkness [9]. Since the common level of auxin might fail to meet the specific requirements for wheat with various genetic backgrounds, in the present study six concentration of DIC, PIC and 2,4 D were chosen to evaluate the morphogenic response of einkorn immature embryos. In contrast to the numerous observations reporting that auxin could be used as a sole biological inductor for effective morphogenic response in polyploid wheats [14, 16, 19, 20, 22–23, 27–29, 31], the results presented here testify that it couldn’t be regarded as a key factor in enhancing the regeneration potential capacity of einkorn cultured tissues. Since various tested levels of auxins were equally ineffective in the present study, the genetic components of einkorn are thought to be prevailing over the exogenous auxin in controlling of *in vitro* response. Indeed, only the use of combination of auxins with other PGRs, primarily DAM, and then phenyl-urea cytokinins, could overcome the genotypic recalcitrance of einkorn.

The inclusion of DAM substantially increased the frequency of morphogenic structures formation in the case when the immature embryos of einkorn were cultured on the medium supplemented with various types of synthetic auxins (Table 1, Table 4). DAM is well known since 1960s as a plant growth retardant that controls stem growth, plant size and fruit ripening [43]. *In vivo*, this compound is proposed to inhibit 2-oxoglutarate oxygenases involved in gibberellin biosynthesis in plants [44] as well as to inhibit the ethylene production by blocking the conversion of methionine to aminocyclopropane-1-carboxylic acid in fruits [45]. In line with this DAM acted in einkorn similarly to various plant growth retardants and ethylene inhibitors, which previously were found to stimulate somatic embryogenesis and plant regeneration in cereals including barley [46], maize [47] and rice [48]. DAM was successfully used in combinations with various PGRs to promote somatic embryogenesis and plant regeneration in potato [49], lily [50] and loblolly pine [51]. Similarly to the results obtained here for diploid einkorn, DAM in combination with various auxin-like substances increased the embryogenic competence of immature embryo culture of eleven hexaploid *T*.*aestivum* varieties [34]. At the same time such the phenomenon as a low frequency of induced structures conversion into the viable plants was a sensitive problem for einkorn cultures induced in the presence of DAM. Evidently the leading role in observed growth delay belongs to DAM, as the higher the concentration of retardant was used previously for hexaploid wheat; the more excessive conversion of morphogenic callus into dormant non-regenerative clumps was observed [34].

Our histological examination revealed that cells of induced nodules turned to heterogeneous and varied in shape, size and the degree of vacuolization starting from the second week of cultivation; the accumulation of storage preserves was clearly observed in many of them (Fig 4). The similar observation for abundant starch and protein accumulation was found in the scutellum of hexaploid wheat embryos developing in vitro from isolated zygotes [52]. In agreement with this, the “dormant” structures formed in the presence of DAM showed visual similarity with scutellum of zygotic embryos being hard, white and non translucent. It is interesting, that significant starch deposition was also detected in durum wheat somatic embryos due to a supplementation of AgNO_3_ [35], the substance that acts in vitro similarly to DAM, namely inhibits the production of ethylene, one of the plant growth regulators involved directly into fruit and seed maturation. Therefore the exact mode of action of DAM is not fully understood, apparently it induces a various side biosynthesis pathways. Plant growth retardants are known to alter endogenous levels of sterols, carotenoids, cytokinins, brassinosteroids and abscisic acid [43], thus cause various side effects in vitro [51]. Although the observed correlations do not necessarily imply causal relationship with side biosynthesis pathways, we suggested that DAM, been plant growth retardant, above certain concentrations can act as stress-inducer that promote mechanisms of differentiation and maturation normally observed during in planta embryonic development.

At the same time, among ‘dormant-like’ masses of cells, small isodiometric cells with dense cytoplasm were still able to form active meristematic centers during both induction and maturation phases in einkorn cultures. Hence, the remaining competent cells–mainly in the superficial layers–proliferate by generating somatic embryos or secondary organogenic nodular structures, which later will be able to turn into green plants, especially when regeneration medium was supplemented with cytokinin-like substance, namely TDZ. Interestingly, that in conifers DAM has demonstrated less negative side effects and contributed to initiation of somatic embryogenesis more effectively versus the other tested GA inhibitors [51]. Considering that DAM belongs to the group of GA inhibitors that alters GA biosynthesis late in the biosynthetic pathway [45], Pullman et al. [51] suggested that the side effects of DAM could be reduced compared to the other GA inhibitors. All those observations suggest the complexity of direct and non-direct action of DAM in tissue culture; however, a more detailed study is required to clarify the molecular mechanisms of its multifunctional effects. The practical conclusion from the results presented here, is that DAM in combination with various PGRs is capable to improve significantly the efficiency of morphogenesis in einkorn that has low regeneration ability.

Basically plant regeneration in immature zygotic embryo cultures of polyploid wheat is followed by transfer of induced embryogenic/organogenic structures onto the medium lack of plant growth regulators for embryo maturation/shoot induction and conversion into plantlets [13–14, 16, 22–23, 27, 30–31, 35]. In cereal tissue cultures displaying low conversion into plantlets, the regeneration can be increased by the addition of cytokinins to maintenance or regeneration medium [32]. In the present report the apparent increase of the regeneration ability of induced morphogenic structures was observed due to application of TDZ during regeneration phase. This action allows doubling the average number of shoots per callusing immature embryo and partially improves the overall tissue culture efficiency of einkorn. Similar high shoot induction potential for TDZ was observed in bread, durum, emmer and kiharae wheat, when it was compared with the other plant growth regulators added to regeneration media [27, 31, 36–37]. Nevertheless, in reports dealing with the culture of wheat immature embryos, the optimal choice for cytokinin in regeneration media depended largely on the used genotype, so the difference between the studied variants was not always noticeable [17, 27–28, 31, 36].

Evidences from the previous reports indicate that cytokinins might be directly included into the induction medium in order to stimulate morphogenic response in a number of dicotyledonous species [53]. In cereals tissue culture, however, the molecular link of cytokinin with callusogenesis and embryogenesis is not always established [32]. Our preliminary experiments showed that the presence of cytokinins (BA and TDZ) in callus induction media along or in combination with auxin (DIC) induced oxidation of cultured immature embryos, arrested the callus formation and did not affect the morphogenesis in einkorn (data are not shown). Nevertheless, there are few examples, when combinations of various types of plant growth regulators have been reported to play regulatory roles on the embryogenic callus production and subsequent shoot regeneration in cereals. In wheat, for instance, three PGRs (auxin and two cytokinins) were successfully used by Chauhan et al. [27] to stimulate somatic embryogenesis from mature and immature embryos of *T*.*aestivum*, *T*.*durum* and *T*.*diccocum*. Treatment of immature embryos with a mix of two auxins and one cytokinin promoted the rapid induction of direct somatic embryogenesis in various cereals species, including wheat, ray, barley and oats [12]. A combination of auxin, cytokinin and Zearalenone, a fungus-derivative substance displaying regulatory ability to control plant development, recently demonstrated to be suitable for stimulation of somatic embryogenesis in recalcitrant genotype of *T*.*aestivum* [54]. Our experiment for the first time has demonstrated that the adjustment of tissue culture media by combination of different types of PGRs, such as synthetic auxin, GA biosynthesis inhibitor DAM and phenylurea derivative cytokinin, could result in higher success for morphogenesis and shoot formation in cereals, particularly in *T*.*monococcum*. The stability of positive synergic effect even if various auxin-like substances or urea-type cytokinins were combined with DAM indicates the uniform mode of such combination. Most likely, it ensures the switching-on genetic programs responsible for regulating processes of cell division and formation of morphogenic structures, either directly or indirectly.

Our findings are consistent with the observations on tissue culture of recalcitrant dicots and monocots, indicating that synthetic phenylurea derivatives (TDZ and CPPU) demonstrated higher activities than those of adenine type derivatives, such as BA, Kinetin, ZEA [32, 55]. Recent evidence has indicated that TDZ and CPPU, besides the cytokinin activity, induce metabolic cascade that modifies a primary signaling event, accumulation and transfer of various endogenous plant signals such as auxin and melationin, a system of secondary messengers and a concurrent stress response [53]. Despite the fact that both TDZ and CPPU were introduced into plant cell and tissue culture in the mid-80s, TDZ currently is much more widely used as plant growth regulator than CPPU. Recently it was shown that CPPU treatment induces variations in many proteins involved in plant growth and development of bread wheat seedling [56]. However, as far as we know CPPU was never used in wheat tissue culture either for induction of embryogenesis or for stimulation of plant regeneration. Although both CPPU and TDZ supported morphogenesis equally effective in combination with DIC and DAM, the presented results, however, indicates that TDZ is the better choice for immature embryo culture of einkorn since it induced significantly more plantlets than CPPU.

In general, the presence of urea-type cytokinin caused the formation of first plantlets as early as on 35–40 days after the culture initiation (Fig 1H and 1K). Similarly multiple shoots were observed to arise directly from mature zygotic embryo explant of polyploid wheats, barley and triticale, with no evidence of any intermediate callus tissue when Phenyl-urea cytokinin was used solely for stimulation of morphogenic response [37]. It is interesting that in the present study the direct shoot formation from einkorn explants has never been observed when the induction medium was lacking of urea cytokinin. That suggests a critical role of TDZ and CPPU on the timing of the morphogenic structures formation and plantlets development in cultures of einkorn when various combinations of PGRs were used. Taking into account a short period when the process was observed in the presence of Phenyl-urea cytokinins, our results suggest that the direct somatic embryogenesis and shoot formation has been occurred. Histological observations confirmed that the presence of urea-type cytokinin intercepts the maturation pattern of differentiated cells and thus speed up the formation of first plantlets. On the other hand, the development of concomitant secondary morphogenic callus was also observed. This confirms the previous observations which have shown that the somatic embryogenesis and organogenesis are both morphogenetic processes that may occur *in vitro* in the same cereal cultures [15, 39–41]. Despite the fact of variation in morphogenic response, asynchronous development of both organogenesis and embryogenesis in the same einkorn cultures indicate the complexity of biological activity provided by tested PGRs combinations.

Susceptibility of cells which are potentially competent for morphogenic response to genetic programming and reprogramming by external factors is largely depended on the development stage of plant tissue used for initiation. In cereals a close relation between the embryogenic competence and the morphological stages of embryo development has been known for decades [16, 21–22, 29, 57–58]. The reason that explants of the different developmental stage exhibit different capability for somatic embryogenesis and plant regeneration is presumably associated with the endogenous hormones levels in donor plant tissue, notably in kernels prior to embryo excision culture [59]. Respectively to the initial endogenous levels of PGRs, einkorn explants of the same physiological stage resulted in the substantial variation when single or multiple synthetic PGRs were used as external factors to promote morphogenic response. With an appropriate callus induction medium consisted of three PGRs, an optimal developmental stage for einkorn was found to be partially transparent immature embryo in size of about 1.0 mm. In bread and durum wheat the size and the color of the embryos is a very good practical indicator for the right developmental stage to culture initiation. Generally, immature embryos that are milky translucent in color and between 0.75 and 1.5 mm in length are optimal for plant regeneration [16, 18, 21]. Einkorn, in contrast to polyploid wheat, has rather small kernels, and somewhat smaller embryos at the stage of isolation. Although both small- and big-sized explants are equally suited for the reasonable induction of morphogenesis using the combination of three PGRs, some technical reasons are to be considered. Evidently, the safe dissection of small-sized embryos was practically more difficult, so in case of occasional injury they may not be capable of callus formation. At the other hand, the big-sized explants demonstrated much higher precocious germination (69%), than intermediate ones (39%), therefore some additional manipulations *in vitro* were required to remove developing embryo axis during the callus induction phase.

## Conclusions

In conclusion, efficient and reliable plant regeneration protocol for immature embryos of diploid einkorn wheat through somatic embryogenesis and organogenesis has been successfully established. The presented here protocol allows the rapid production of plenty of regenerants within 75 days starting from initial explant, without the signs of morphological abnormalities. The protocol is rather simple and practical, since it does not require a plenty of culture media (consisted of a high number of components) to be changed during the cultivation. To our knowledge, this is the first report in which combinations of three PGRs such as auxin, cytokinin and GA-biosynthesis inhibitor DAM, displaying distinctive biological activities, have been simultaneously used for promoting a high rate of morphogenesis in wheat. In any event the efficiency of induction either of embryogenic/morphogenic structures or plant regeneration outperforms all known reports dealing with *in vitro* tissue culture of einkorn and other diploid wheat species. Although in the present study the critical balance between different PGRs was established for einkorn only, we assume that the proposed strategy might be further successfully applied to other recalcitrant wheat genotype and species to efficiently regulate morphogenesis and plant formation.

## Supporting information

S1 Table The influence of various concentrations of auxins on morphogenesis and plant regeneration from immature embryos of einkorn (*Tritcum monococcum* L.). All cultures were initiated within 30 days on callus induction medium supplemented with the tested auxin with the subsequent two subcultivations for 15 and 30 days on the regeneration medium lack of PGRs. Means having the same letter in the column has no significant differences according to Duncan’s multiple range test (*P* < 0.05); ***ns*** indicates that there is no significant difference between auxin types at 5% level according to LSD test.
(DOCX)
