# Supplementary material for: Protocol for efficient regulation of in vitro morphogenesis in einkorn (Triticum monococcum L.), a recalcitrant diploid wheat species
Source: PLoS One. 2017 Mar 8;12(3):e0173533. doi: 10.1371/journal.pone.0173533 (PMC5342269; doi:10.1371/journal.pone.0173533)
Supplement: S1 Table — All cultures were initiated within 30 days on callus induction medium supplemented with the tested auxin with the subsequent two subcultivations for 15 and 30 days on the regeneration medium lack of PGRs. Means having the same letter in the column has no significant differences according to Duncan’s multiple range test (P < 0.05); ns indicates that there is no significant difference between auxin types at 5% level according to LSD test. (DOCX) [file pone.0173533.s001.docx]

| Auxin | Concentration (mg L^-1^) | Callus induction (%) | Morphogenic callus formation (%) | Percentage of regenerating calli (%) | No. of plantlets per regenerating calli | | No. of plantlets per callus |
| --- | --- | --- | --- | --- | --- | --- | --- |
| DIC | 1 | 90.29 a | 2.15 c | 1.08 ab | 2.00 ef | | 0.02 cd |
|  | 2 | 98.17 a | 4.67 abc | 1.87 ab | 5.00 abcde | | 0.09 bcd |
|  | 3 | 96.67 a | 7.76 a | 5.17 a | 4.33 bcdef | | 0.22 a |
|  | 4 | 98.35 a | 7.56 ab | 5.04 a | 4.00 bcdef | | 0.20 ab |
|  | 5 | 98.32 a | 2.56 bc | 1.71 ab | 7.00 abc | | 0.12 abc |
|  | 6 | 95.83 a | 3.48 abc | 2.61 ab | 7.33 abc | | 0.19 ab |
|  | mean | 96.27 ns | 4.07 ns | 2.91 ns | | 4.94 ns | 0.14 ns |
| PIC | 1 | 96.92 a | 1.59 c | 0.00 b | 0.00 f | | 0.00 d |
|  | 2 | 94.70 a | 4.90 abc | 4.90 a | 2.29 def | | 0.11 abc |
|  | 3 | 94.03 a | 3.97 abc | 3.17 ab | 4.75 abcde | | 0.15 ab |
|  | 4 | 95.65 a | 3.79 abc | 3.03 ab | 6.00 abcde | | 0.18 ab |
|  | 5 | 99.13 a | 2.63 bc | 2.63 ab | 6.67 abcd | | 0.18 ab |
|  | 6 | 96.52 a | 3.60 abc | 3.60 ab | 5.75 abcde | | 0.21 ab |
|  | mean | 96.16 ns | 3.41 ns | 2.89 ns | 4.24 ns | | 0.14 ns |
| 2,4-D | 1 | 94.23 a | 2.04 c | 2.04 ab | 8.00 ab | | 0.16 ab |
|  | 2 | 90.97 a | 3.55 abc | 2.13 ab | 9.00 a | | 0.19 ab |
|  | 3 | 94.16 a | 4.14 abc | 2.76 ab | 5.25 abcde | | 0.14 ab |
|  | 4 | 90.91 a | 0.71 c | 0.71 ab | 3.00 cdef | | 0.02 cd |
|  | 5 | 96.36 a | 0.00 c | 0.00 b | 0.00 f | | 0.00 d |
|  | 6 | 91.96 a | 0.97 c | 0.00 b | 0.00 f | | 0.00 d |
|  | mean | 93.10 ns | 1.90 ns | 1.27 ns | 4.21 ns | | 0.13 ns |
